# Global epidemiology of invasive meningococcal disease

**DOI:** 10.1186/1478-7954-11-17

**Published:** 2013-09-10

**Authors:** Rabab Z Jafri, Asad Ali, Nancy E Messonnier, Carol Tevi-Benissan, David Durrheim, Juhani Eskola, Florence Fermon, Keith P Klugman, Mary Ramsay, Samba Sow, Shao Zhujun, Zulfiqar A Bhutta, Jon Abramson

**Affiliations:** 1 Department of Pediatrics and Child Health, Division of Women and Child Health, Aga Khan University, Stadium Road, Karachi, Pakistan; 2 Centers for Disease Control and Prevention, Atlanta, GA, USA; 3 Immunisation, Vaccines and Biologicals, World Health Organisation, Geneva, Switzerland; 4 School of Medicine and Public Health, University of Newcastle, Callaghan, Australia; 5 Health Protection, Hunter New England Area, Wallsend, Australia; 6 Finnish National Institute for Health and Welfare (THL), Helsinki, Finland; 7 International vaccination working group, Médécins Sans Frontières, Paris, France; 8 Global Health, Rollins School of Public Health, Emory University, Atlanta, GA, USA; 9 Respiratory and Meningeal Pathogens Research Unit, University of Witwatersrand and Medical Research Council, Johannesburg, South Africa; 10 Immunisation Department at the Health Protection Agency, Centre for Infections in Colindale, London, UK; 11 Center for Vaccine Development, Ministry of Health, Bamako, Mali; 12 School of Medicine, University of Maryland, Baltimore, MD, USA; 13 Institute for Communicable Disease Control and Prevention, Beijing, People’s Republic of China; 14 Wake Forest School of Medicine, Winston-Salem, NC, USA

**Keywords:** Meningococcus, Neisseria meningitidis, Invasive meningococcal disease, Meningitis, Epidemiology, Meningitis belt

## Abstract

*Neisseria meningitidis* is one of the leading causes of bacterial meningitis globally and can also cause sepsis, pneumonia, and other manifestations. In countries with high endemic rates, the disease burden places an immense strain on the public health system. The worldwide epidemiology of invasive meningococcal disease (IMD) varies markedly by region and over time. This review summarizes the burden of IMD in different countries and identifies the highest-incidence countries where routine preventive programs against *Neisseria meningitidis* would be most beneficial in providing protection. Available epidemiological data from the past 20 years in World Health Organization and European Centre for Disease Prevention and Control collections and published articles are included in this review, as well as direct communications with leading experts in the field. Countries were grouped into high-, moderate-, and low-incidence countries. The majority of countries in the high-incidence group are found in the African meningitis belt; many moderate-incidence countries are found in the European and African regions, and Australia, while low-incidence countries include many from Europe and the Americas. Priority countries for vaccine intervention are high- and moderate-incidence countries where vaccine-preventable serogroups predominate. Epidemiological data on burden of IMD are needed in countries where this is not known, particularly in South- East Asia and Eastern Mediterranean regions, so evidence-based decisions about the use of meningococcal vaccines can be made.

## Introduction

*Neisseria meningitidis* is one of the leading causes of bacterial meningitis globally and can also cause sepsis, pneumonia, and other localized infections. There are 12 serogroups, but the majority of invasive meningococcal infections are caused by organisms from the A, B, C, X, Y, or W-135 serogroups. The annual number of invasive disease cases worldwide is estimated to be at least 1.2 million, with 135,000 deaths related to invasive meningococcal disease (IMD) [1,2]. In countries with high endemicity, the disease burden places an immense strain on the public health system. The risk of long-term disabling sequelae, including cognitive deficit, bilateral hearing loss, motor deficit, seizures, visual impairment, hydrocephalus, and loss of limbs due to tissue necrosis, are highest in low-income countries, where the burden of bacterial meningitis is greatest [3].

To combat IMD, many industrialized countries have included different formulations of meningococcal vaccine in their routine immunization programs. A vaccine against serogroup A has recently been introduced in the African meningitis belt, an area extending from Senegal in the west to Ethiopia in the east [4,5]. However, meningococcal vaccines remain underutilized globally, particularly in resource-limited countries outside the African meningitis belt. To provide cost effective recommendations about the use of meningococcal vaccines, the country-specific burden of IMD must be established [6]. A comprehensive review of IMD incidence, including all countries with at least a basic surveillance infrastructure reporting IMD cases, was conducted. The review provides the most recently published attack rates, predominant serogroups, and at-risk groups from over 80 countries and organizes the data according to priority groups for vaccine intervention.

## Methods

### Search strategy and selection criteria

Our sources for the epidemiological data included the National Library of Medicine (PubMed), the World Health Organization (WHO) website of the Weekly Epidemiological Record, and the European Centre for Disease Prevention and Control. We searched PubMed with the following key terms: (“Neisseria meningitidis”) OR (“meningococcal”) OR (“meningococcemia”) OR (“meningococcus”). The search was limited to studies of humans, studies published in English, and dates of publication from January 1, 1990, to December 31, 2010. The initial search yielded 5,336 results from which studies were excluded based on exclusion criteria below. In addition, data were obtained and included from WHO publications in the *Weekly Epidemiological Record* (WER) for the latest figures from 14 African meningitis belt countries. The European Union Invasive Bacterial Infections Surveillance Network (EU-IBIS), which is maintained by the European Centre for Disease Prevention and Control, was accessed for updated figures for European countries and these data were also included. We searched reference lists in all identified articles for additional articles, and reviewed abstracts and titles and selected studies if it seemed they included aspects of meningococcal epidemiology. From the above literature search we excluded generic worldwide estimates (except to identify original data references), or studies that were limited to immunology, drug resistance, or other non-epidemiological factors.

### Organization of data

**Figure 1:**
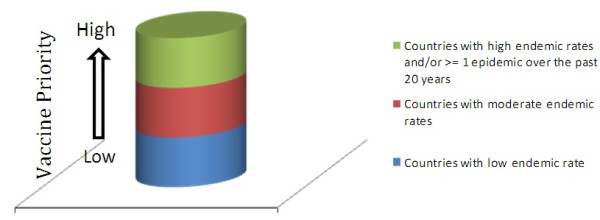
A categorization of countries according to IMD attack rates.

The WHO definition of a meningococcal disease epidemic (>100 cases/100,000 population/year) applies specifically to the meningitis belt. Other countries rarely experience epidemics with these high attack rates. We classified countries according to the level of endemic meningococcal disease as “high,” “moderate,” and “low” endemicity (Figure 1). This classification is based on country-specific epidemiological data with pre-defined cutoffs of high, moderate, and low endemicity categories and was used by the WHO’s Strategic Advisory Group of Experts on Immunizations (SAGE) in its recently updated recommendations on the use of meningococcal vaccines [7].

## Results

### Epidemiology of meningococcal disease at country level

Data on incidence of meningococcal disease are presented below in Tables 1, 2 and 3. Countries are grouped into priority regions according to the definitions above, using national and published data from the last 20 years. Countries not listed in the table have insufficient available IMD epidemiological data to permit accurate classification.

**Table 1:** Countries with high endemic rates (>10 cases/100,000 population) and/or > =1 epidemic over the last 20 years

**Country**	**Year**	**Incidence/100,000 population**	**Predominant serogroup**	**Source**	**Comments**
**African Region**					
**Angola**	1994–2000	19–230	*	[8]	
**Benin**	1980–1999	6–57		[9,10]	
**Burkina Faso**	2004–2009	26–187		[11,12]	
**Burundi**	1980–1999	158		[9]	
**Cameroon**		1–224		[13]	
**Centrafique**	2004–2009	3 3–19.4		[12]	
**Chad**		9.6–15.9		[12]	
**Cote de Ivoire**	1980–1999	0-6		[14]	Despite its relatively low attack rate, Cote de Ivoire is included in this table due to its location in the meningitis belt
**Ethiopia**		0–104	A	[9,10]	
**Gambia**		4–165		[9,15]	
**Ghana**		0–108		[9,15]	
**Guinea**		0–17		[12]	
**Guinea Bissau**		0–133		[9]	
**Kenya**	1990	267		[16]	
**Mali**	2004–2009	2.6–12.9		[12]	
**Mauritania**	1980–1999	0–14		[9]	
**Namibia**		4–165		[9]	
**Niger**	2004–2009	7.8–90.7		[17]	
**Nigeria**		0.7–52.6		[12]	
**RD Congo**		7.3–23.7		[12]	
**Rwanda**	1980–1999	0–28		[9]	
**Senegal**		0–53			Incidence >50 in 1983
**Tanzania**	1980–1999	0–19			
**Togo**	2004–2009	6–13.2		[12]	
**Uganda**	1980–1999	0–18		[14]	
**Eastern Mediterranean Region**					
**Sudan**	2008	*	A	[12]	Despite lack of data Sudan is included in this table due to its location in the meningitis belt
**Saudi Arabia**	2000		A, W-135	[18]	225 cases in month after 2000 Hajj season. Data from Saudi Arabia mostly includes cases from the Hajj season.
**European Region**					
**No country in this region is in the high rate category**					
**Region of the Americas**					
**Uruguay**	2001	30 (pre-vaccine)	B	[19]	Vaccine comprising serogroup C capsular polysaccharide and the outer membrane vesicles of serogroup B meningococcus was used
		1.6 (post-vaccine)			
**South-East Asia Region**					
**No country in this region is in the high rate category**					
**Western Pacific Region**					
**New Zealand**	1991–2000	17.4 (pre-vaccine)	B	[20]	An OMV vaccine for Serogroup B was introduced in 2004
		2.6 (post-vaccine)			
**Mongolia**	1994–1995	80-90	A	[21]	

* Data not available.

**Table 2:** Countries with moderate endemic rates (2–10 cases/100,000 population per year)

**Country**	**Year**	**Incidence/100,000 population**	**Predominant serogroup**	**Source**	**Comments**
**African Region**					
**South Africa**	2000–2005	0.8–4	B in Western Cape	[22]	
**Eastern Mediterranean Region**					
**No country in this region is in the moderate rate category**					
**European Region**					
**Belgium**	1999–2010	2.9 (pre-vaccine)	B, C	[23,24]	A conjugate vaccine for group C was introduced in 2002
		0.89 (post-vaccine)			
**Denmark**	1999–2010	1.19–3.5	B	[23,24]	
**Greece**		0.49–2.0	C	[23,24]	A conjugate vaccine for group C introduced in 2001 in pediatric population[25]
**Ireland**		14.3 (pre-vaccine)	B, C	[23,24]	A conjugate vaccine for group C was
		2.19 (post-vaccine)			introduced in 2001
**Iceland**		7.6 (pre-vaccine)	B, C	[23,24]	A conjugate vaccine for group C was
		0.6 (post-vaccine)			introduced in 2002
**Lithuania**	2004–2010	1.4–2.6	*	[23,24]	
**Luxemburg**	1999–2010	0.2–5.68	*	[23,24]	
**Malta**	1994–2007	0.8–8.9	B, C	[26]	2 peaks in 2000 and 2006
**Netherland**	1999–2010	3.6 (pre-vaccine)	B, C	[23,24]	A conjugate vaccine for group C was introduced in 2002
		0.86 (post-vaccine)			
**Norway**	1992–2010	0.8–4.6	B	[23,27]	
**Portugal**	2000–2010	0.74–3.0	B, C	[23,28]	
**Spain**	1999–2010	3.52 (pre-vaccine)	B, C	[23,24]	A conjugate vaccine for group C introduced in 2001
		0.88 (post vaccine)			
**Switzerland**	1999–2004	1.16–2.36	C	[24]	A conjugate vaccine for group C introduced in 2005
**Turkey**	1997–2005	0.3–2.2	*	[28]	
**United Kingdom**	1999–2010	5.4 (pre-vaccine)	B, C	[23,24]	A conjugate vaccine for group C introduced in 1999
		1.63 (post vaccine)			
**Region of the Americas**					
**Brazil**	1998–2006	1–4.5	B, now C	[19]	A combined vaccine against serogroup B (OMV) and C (polysaccharide) was introduced in 1990
**Cuba**	1998–2003	3.4-8.5 (pre-vaccine)	B	[29]	A combined vaccine against serogroup B (OMV) and C (polysaccharide) was introduced in 1987
		<1 (post-vaccine)			
**South-East Asia Region**					
**No country in this region is in the moderate rate category**					
**Western Pacific Region**					
**Australia**	1995–2006	3.5–7.9 (pre-vaccine)	B	[30]	A conjugate vaccine for Serogroup C was introduced in 2003
		1.4 (post-vaccine)			

* Data not available.

**Table 3:** Countries with low endemic rates (<2 case/100,000 population per year)

**Country**	**Year**	**Incidence/100,000 population**	**Predominant serogroup**	**Source**	**Comments**
			**African Region**		
		**No country in this region is in the low rate category**			
			**European Region**		
**Austria**	1999–2010	1.02–1.2	B, C	[23,24]	
**Bulgaria**	2000–2010	0.11–1.1	*	[23,28]	
**Croatia**	1997–2005	0.7–1.3	*	[28]	
**Cyprus**	1997–2010	0.13–1.7	*	[23,28]	
**Czech Republic**	1999–2010	0.57–1.0	B, C	[23,24]	
**Estonia**	2001–2010	0.15–1.6	*	[23,28]	
**Finland**	1999–2010	0.64–1.1	B	[23,24]	
**France**		0.7–1.13	B, C		
**Germany**		0.47–0.73	B, C		
**Hungary**	2004–2010	0.3–0.4	*		
**Italy**	1999–2010	0.25–0.55	B, C		
**Latvia**	2004–2008	0.25–1.03	*		
**Poland**	1999–2010	0.17–0.84	B		
**Serbia**	2000	0.9	*	[28]	
**Slovakia**	2004–2010	0.59–0.9	*	[23,24][23,24]	
**Slovenia**	1999–2010	0.3–1.2	*		
**Sweden**	2004–2010	0.5–0.7	B, C		
			**Eastern Mediterranean Region**		
		**No country in this region is in the low rate category**			
			**Region of the Americas**		
**Argentina**	2009	0.6	B	[31]	
**Canada**	1985–2006	1.4 (pre-vaccine)	C	[32,33]	Vaccination in 2001–2 in all provinces
		0.4 (post-vaccine)			
**Chile**	1998–2006	0.8	B	[19]	
**Columbia**		0.3	Y		
**Mexico**		0.1	C		
**USA**	2000–2009	0.8 (pre-vaccine)	Equal B, C,Y	[34]	Routine vaccination program started in 2005
		0.3 (post-vaccine)			
**Venezuela**		0.3	Y	[19]	
			**South-East Asia Region**		
**Korea**	2002–2008	<0.1		[35]	
**Thailand**	2007–2008	<0.1		[36]	Higher in <5 year olds
			**Western Pacific Region**		
**China**	2000 onward	<0.2	A, C	[37,38]	
**Japan**	1999–2004	<0.02	*	[39]	
**Philippines**	2004–2008	<0.1	A	[1]	
**Singapore**	2005–2009	0.1–0.2		[36,40]	25/100,000 in Hajj pilgrims in 2000 [41]
**Taiwan**	2000–2001	0.1–0.2	A	[42]	
		**Eastern Mediterranean Region**			
		**No country in this region is in the low rate category**			

*Data not available.

**Figure 2:**
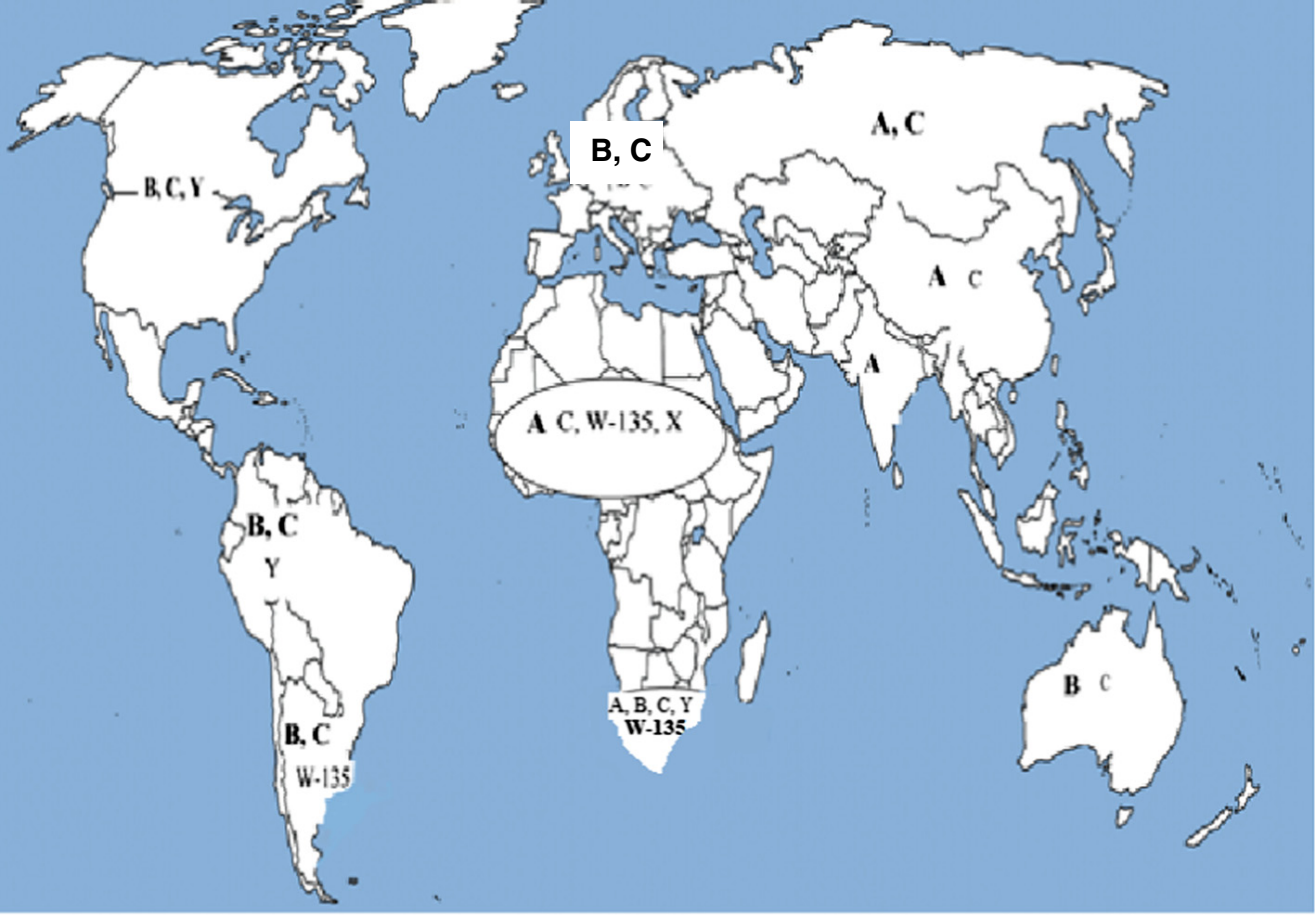
Distribution of common and predominant meningococcal serogroups by region. Predominant strains are highlighted in bold text.

Significant gaps in data limit description of IMD epidemiology in some parts of the world. In many countries with IMD surveillance, extensive targeted vaccine development and increasing coverage has decreased the burden of disease. In some endemic countries without vaccination, high IMD attack rates continue. The specific regional epidemiology is summarized in Figure 2 and described in terms of WHO regions below.

### African region

The African Meningitis Belt, originally characterized by Lapeysonnie in 1963 [5] and modified in 1987, has the highest annual incidence of meningococcal disease in the world with superimposed frequent epidemics that constitute a major public health burden. Outbreaks in the sub-Saharan region coincide with the dry season, which has led to a hypothesis for the potential role of low humidity and seasonal dust-wind blowing from the Sahara (the Harmattan) in damaging the mucosa and producing irritant coughing that aids transmission [34,43].

Twenty-five countries in the African region with an extremely high incidence of meningococcal disease constitute the meningitis belt. To rapidly detect the frequent epidemics, a strong surveillance system exists that monitors the number of cases on an ongoing basis for rapid response. This region has recently benefitted from a major alliance of international health bodies that have developed and are deploying an affordable and effective vaccine against serogroup A meningococcus, which causes the majority of disease in this area, at population level [44].

Serogroup X, previously a rare cause of sporadic meningitis, has been responsible for outbreaks between 2006 and 2010 in Kenya, Niger, Togo, Uganda, and Burkina Faso, the latter with at least 1,300 cases of serogroup X meningitis among the 6,732 reported annual cases [45].

South Africa is included in the moderate-endemicity group, while other countries in this region do not have adequate data to enable derivation of a population-based estimate of their true incidence rates.

### Eastern Mediterranean region

Sudan and Saudi Arabia have high endemic rates of serogroup A disease, and have also experienced outbreaks in recent years during the hajj season with serogroup W-135. Other countries in this region do not have adequate data to permit a population-based estimate of true incidence, although outbreaks have been reported among returning travelers from Hajj.

### European region

With the exception of a few countries in the eastern part of the European Region, good surveillance data are available from most European countries. Serogroup B and C are responsible for the majority of disease, and implementation of a meningococcal immunization program with adequate vaccine coverage has contributed to decreasing endemic rates so that no country now falls in the high-endemicity group. Fifteen countries from this region are classified as moderate endemicity and 18 as low. Recent epidemiological surveillance indicates an increase of serogroup Y IMD in some parts of Europe, which is now the third most common serogroup after B and C [23].

### Region of the Americas

Uruguay remains the only country from this region to have experienced high rates of IMD within the past 20 years. In 2001, it suffered a peak incidence rate of meningococcal disease due to serogroup B and this prompted the introduction of the Cuban outer membrane vesicle (OMV) B vaccine with good coverage and a sharp decline in incidence in subsequent years. Brazil and Cuba have experienced moderate incidence rates, but have also seen significant benefit from the introduction of meningococcal vaccines in their populations [29]. Argentina, Canada, Chile, Columbia, Mexico, the United States, and Venezuela have experienced low levels of IMD in the timeframe defined by this review. Serogroup Y emerged in Colombia and Venezuala, where it became the common disease-causing serogroup in 2006 [19]. The US has a universal meningococcal vaccine (conjugated quadrivalent vaccine given to adolescents) and Canada also recommends a booster dose in this age group following primary immunization at 12 months of age. Other countries in this region do not have adequate data to allow population-based estimates of their true incidence.

### South-East Asia Region

Korea and Thailand are the only countries from this region with published population-based estimates, which demonstrate low endemic rates. India has experienced repeated serogroup A epidemics, the most recent in 2005, but data are mostly available only from large city centers [46]. Sporadic and incomplete data from India, Bangladesh, Indonesia, Nepal, and Pakistan preclude their classification, and no data are available from Sri Lanka [36].

### Western Pacific

New Zealand and Mongolia have recorded high IMD endemicity. New Zealand experienced an outbreak of serogroup B disease until an aggressive campaign with the OMV vaccine was initiated in 2004 that has contributed in part to lowering the incidence. Mongolia experienced serogroup A epidemics in the early 1990s. Australia currently experiences predominantly serogroup B disease with moderate attack rates after the introduction of a serogroup C vaccine saw a marked decline in rates of disease due to the C serogroup. China, Japan, Korea, Philippines, Singapore, Taiwan, and Thailand all experience low levels of IMD. Other countries in this region do not have adequate population-based data to allow estimation of their true incidence rates.

### Age-specific attack rates

In many countries with epidemiological data, particularly in Europe and North America, the age distribution of meningococcal disease demonstrates two peaks [6,47-49]. The highest incidence is in infants less than one year of age, and a secondary rise in incidence occurs in adolescents and young adults. In one study in the meningitis belt, the age-incidence did not plateau until the late 20s. Fifteen years of data from Niger show that children under the age of five were more affected during epidemics when compared to non-epidemic years [50]. However, some studies have suggested a shift toward older age groups during epidemics [51].

The widespread and rapid use of effective antibiotics has contributed to bringing down the case fatality rate of IMD to between 10 and 20 percent, but it is generally higher in developing countries where access to higher levels of care may be delayed [52]. Despite advances in resuscitative techniques, surgical intervention, and critical care, there is a persistent mortality in the early hours of septicemia due to the rapid progression of disease.

## Conclusion

IMD is a serious illness that can be rapidly progressive with resulting significant morbidity and mortality, even with treatment. Vaccines are available for the majority of serogroups that cause disease and have proven effective in reducing the disease incidence in countries that have applied them at the population level. Maximizing the global impact of these vaccines requires having them made available in regions that have the highest disease incidence.

This review used available data to define the burden of meningococcal disease in different countries. Countries were classified based on the disease endemicity, and available data on the most prevalent serogroups were reviewed to permit evidence-based decisions on meningococcal vaccine use. These data helped inform SAGE’s new recommendations on the use of meningococcal vaccines. Limitations of the review include exclusion of studies that were not published in English and the absence of an indicator of the quality of surveillance used to derive incidence rates. It is crucial that timely surveillance utilizing new molecular epidemiology tools is implemented to obtain epidemiological data on burden of meningococcal disease in countries where these are not known. With the licensing of a new affordable conjugated vaccine against serogroup A (licensed in Africa) and, more recently, a multicomponent serogroup B vaccine in Europe (January 2013), functioning surveillance will be necessary to monitor the impact of these vaccines through direct immunity and herd protection and allow for optimization of vaccination schedules.

## Competing interests

The authors declare that they have no significant competing interests.

## Authors’ contributions

All authors have made active contributions in conceptualizing, drafting, and editing this manuscript. All authors read and approved the final manuscript.

## Authors’ information

This manuscript describes the major findings of the meningococcal vaccine working group commissioned by the Strategic Advisory Group of Experts (SAGE) of the WHO.
